# 1-Dichloro­acetyl-8a-methyl-1,2,3,4,6,7,8,8a-octa­hydro­pyrrolo­[1,2-*a*]pyrimidin-6-one

**DOI:** 10.1107/S1600536812024063

**Published:** 2012-06-02

**Authors:** Shuang Gao, Li-xia Zhao, Fei Ye, Ying Fu, Zhi-yong Xing

**Affiliations:** a College of Science, Northeast Agricultural University, Harbin 150030, People’s Republic of China

## Abstract

In the title compound, C_10_H_14_Cl_2_N_2_O_2_, the five-membered ring adopts an envelope conformation (with the methylene C atom closest to the C—N bridge as the flap), while the conformation of the six-membered ring is close to a twist-boat. In the crystal, mol­ecules are linked by weak C—H⋯O hydrogen bonds, forming chains along the *c*-axis direction.

## Related literature

For general background to 1,5-diaza­bicyclo compounds, see: Fuerst & Lamoureux (1992 ▶); Hutton & Bartlett (2007 ▶); Koptelov *et al.* (2011 ▶); Loriga *et al.* (2007 ▶); Moreland *et al.* (1993 ▶); Taylor *et al.* (2010 ▶). For details of the synthesis, see: Sun & Ye (2010 ▶); Rohr *et al.* (1984 ▶,1986 ▶). For applications of *N*-dichloro­acetyl-1,5-diaza­bicyclo compounds, see: Lamour­eux & Rusness (1992 ▶); Hatzios & Burgos (2004 ▶).
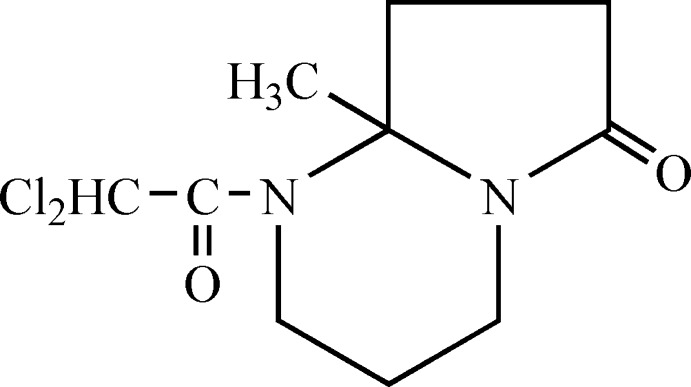


## Experimental

### 

#### Crystal data

C_10_H_14_Cl_2_N_2_O_2_
*M* *_r_* = 265.13
Orthorhombic, 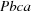

*a* = 10.312 (2) Å
*b* = 14.997 (3) Å
*c* = 15.666 (3) Å
*V* = 2422.7 (8) Å^3^
*Z* = 8
Mo *K*α radiation
μ = 0.52 mm^−1^
*T* = 293 K
0.23 × 0.19 × 0.16 mm

#### Data collection

Rigaku R-AXIS RAPID diffractometer
Absorption correction: multi-scan (*ABSCOR*; Higashi, 1995 ▶) *T*_min_ = 0.889, *T*_max_ = 0.922
22128 measured reflections
2770 independent reflections
2376 reflections with *I* > 2σ(*I*)
*R*_int_ = 0.038

#### Refinement

*R*[*F* ^2^ > 2σ(*F* ^2^)] = 0.052
*wR*(*F* ^2^) = 0.156
*S* = 1.11
2770 reflections
146 parameters
H-atom parameters constrained
Δρ_max_ = 0.74 e Å^−3^
Δρ_min_ = −0.36 e Å^−3^

### 

Data collection: *RAPID-AUTO* (Rigaku, 1999 ▶); cell refinement: *RAPID-AUTO*; data reduction: *CrystalClear* (Rigaku/MSC, 2002 ▶); program(s) used to solve structure: *SHELXS97* (Sheldrick, 2008 ▶); program(s) used to refine structure: *SHELXL97* (Sheldrick, 2008 ▶); molecular graphics: *SHELXTL* (Sheldrick, 2008 ▶); software used to prepare material for publication: *SHELXL97*.

## Supplementary Material

Crystal structure: contains datablock(s) I, global. DOI: 10.1107/S1600536812024063/yk2055sup1.cif

Structure factors: contains datablock(s) I. DOI: 10.1107/S1600536812024063/yk2055Isup2.hkl

Supplementary material file. DOI: 10.1107/S1600536812024063/yk2055Isup3.cml

Additional supplementary materials:  crystallographic information; 3D view; checkCIF report

## Figures and Tables

**Table 1:** Hydrogen-bond geometry (Å, °)

*D*—H⋯*A*	*D*—H	H⋯*A*	*D*⋯*A*	*D*—H⋯*A*
C1—H1⋯O2^i^	0.98	2.15	3.115 (2)	168
C3—H3*B*⋯O2^i^	0.97	2.55	3.502 (3)	169

Symmetry code: (i) 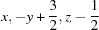.

**Fig. 1.**
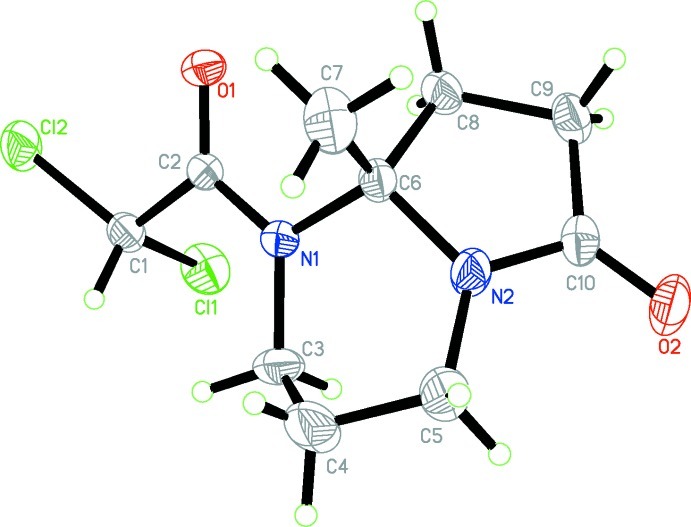
Molecular structure of the title compound with the atom-labelling scheme. Displacement ellipsoids are drawn at the 30% probability level.

**Fig. 2.**
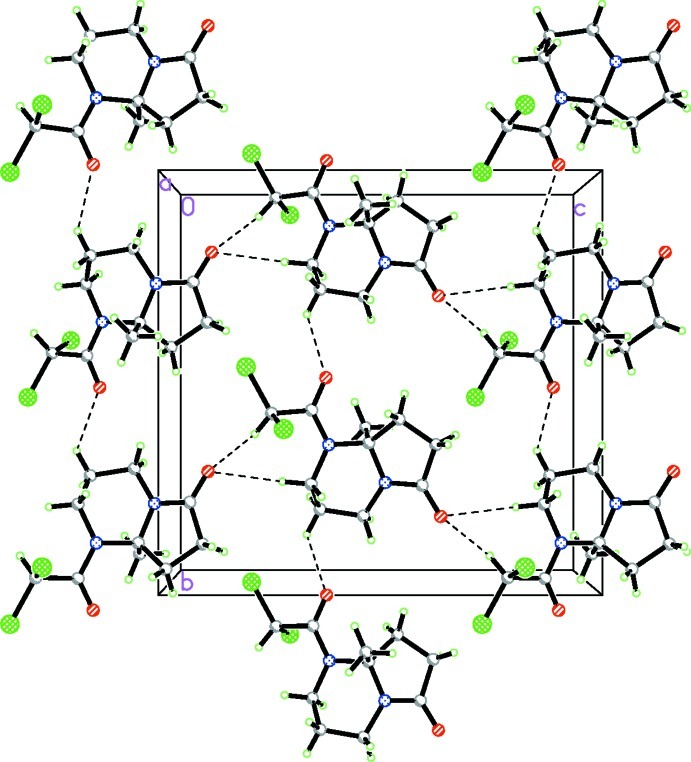
A partial packing view showing hydrogen bonds.

## supplementary crystallographic information

### Comment

Diazabicyclo derivatives are extremely important synthetic intermediates in the syntheses of compounds with potential high biological activity (Fuerst & Lamoureux, 1992; Loriga *et al.*, 2007; Hutton & Bartlett, 2007; Taylor *et al.*, 2010). N-dichloroacetyl-1,5-diazabicyclo compounds have been investigated for usage as herbicide safeners which protect crops from the injury by herbicides (Lamoureux & Rusness, 1992; Hatzios & Burgos, 2004). As a part of our ongoing investigation on the diazabicyclo derivatives we have determined the crystal structure of the title compound.

The molecular structure of the title compound is shown in Fig. 1. In the crystal, molecules are linked by weak intermolecular C—H···O hydrogen bonds, forming chains along the *c* direction (Fig. 2).

### Experimental

The title compound was prepared according to the literature procedure (Sun & Ye, 2010). The single crystal suitable for X-ray structural analysis was obtained by slow evaporation of a solution in the mixture of petroleum ether and ethyl acetate at room temperature.

### Refinement

All H atoms were initially located in a difference Fourier map. The C—H atoms were then constrained to an ideal geometry, with C—H distances of 0.96/0.98 Å, and with U_iso_(H) = 1.2/1.5 *U*_eq_(C).

### Crystal data

**Table:**

C_10_H_14_Cl_2_N_2_O_2_	*F*(000) = 1104
*M**_r_* = 265.13	*D*_x_ = 1.454 Mg m^−^^3^
Orthorhombic, *P**b**c**a*	Mo *K*α radiation, λ = 0.71073 Å
Hall symbol: -P 2ac 2ab	Cell parameters from 9134 reflections
*a* = 10.312 (2) Å	θ = 3.2–27.6°
*b* = 14.997 (3) Å	µ = 0.52 mm^−^^1^
*c* = 15.666 (3) Å	*T* = 293 K
*V* = 2422.7 (8) Å^3^	Block, colourless
*Z* = 8	0.23 × 0.19 × 0.16 mm

### Data collection

**Table:**

Rigaku R-AXIS RAPID diffractometer	2770 independent reflections
Radiation source: fine-focus sealed tube	2376 reflections with *I* > 2σ(*I*)
Graphite monochromator	*R*_int_ = 0.038
Detector resolution: 10 pixels mm^-1^	θ_max_ = 27.5°, θ_min_ = 3.0°
ω scan	*h* = −13→11
Absorption correction: multi-scan (*ABSCOR*; Higashi, 1995)	*k* = −19→19
*T*_min_ = 0.889, *T*_max_ = 0.922	*l* = −20→20
22128 measured reflections	

### Refinement

**Table:**

Refinement on *F*^2^	Primary atom site location: structure-invariant direct methods
Least-squares matrix: full	Secondary atom site location: difference Fourier map
*R*[*F*^2^ > 2σ(*F*^2^)] = 0.052	Hydrogen site location: inferred from neighbouring sites
*wR*(*F*^2^) = 0.156	H-atom parameters constrained
*S* = 1.11	*w* = 1/[σ^2^(*F*_o_^2^) + (0.097*P*)^2^ + 0.5072*P*] where *P* = (*F*_o_^2^ + 2*F*_c_^2^)/3
2770 reflections	(Δ/σ)_max_ = 0.001
146 parameters	Δρ_max_ = 0.74 e Å^−^^3^
0 restraints	Δρ_min_ = −0.36 e Å^−^^3^

### Special details

**Table:**

Geometry. All s.u.'s (except the s.u.'s in the dihedral angle between two l.s. planes) are estimated using the full covariance matrix. The cell s.u.'s are taken into account individually in the estimation of s.u.'s in distances, angles and torsion angles; correlations between s.u.'s in cell parameters are only used when they are defined by crystal symmetry. An approximate (isotropic) treatment of cell s.u.'s is used for estimating esds involving l.s. planes.
Refinement. Refinement of F^2^ against ALL reflections. The weighted R-factor wR and goodness of fit S are based on F^2^, conventional R-factors R are based on F, with F set to zero for negative F^2^. The threshold expression of F^2^ > 2sigma(F^2^) is used only for calculating R-factors(gt) etc. and is not relevant to the choice of reflections for refinement. R-factors based on F^2^ are statistically about twice as large as those based on F, and R- factors based on ALL data will be even larger.

### Fractional atomic coordinates and isotropic or equivalent isotropic displacement parameters (Å^2^)

**Table:**

	*x*	*y*	*z*	*U*_iso_*/*U*_eq_	
C1	0.44507 (19)	0.93130 (12)	0.76600 (12)	0.0425 (4)	
H1	0.4067	0.8855	0.7293	0.051*	
C2	0.36708 (16)	0.93818 (10)	0.84965 (10)	0.0356 (3)	
C3	0.3554 (3)	0.77230 (13)	0.84857 (16)	0.0717 (8)	
H3A	0.4348	0.7471	0.8712	0.086*	
H3B	0.3654	0.7784	0.7873	0.086*	
C4	0.2472 (4)	0.71165 (18)	0.86658 (17)	0.0951 (11)	
H4A	0.2620	0.6552	0.8380	0.114*	
H4B	0.1675	0.7370	0.8444	0.114*	
C5	0.2331 (3)	0.69585 (16)	0.96139 (15)	0.0778 (8)	
H5A	0.1427	0.6854	0.9753	0.093*	
H5B	0.2824	0.6435	0.9779	0.093*	
C6	0.27314 (17)	0.86362 (11)	0.97259 (10)	0.0388 (4)	
C7	0.1334 (2)	0.89612 (19)	0.96606 (15)	0.0643 (6)	
H7A	0.0849	0.8564	0.9301	0.096*	
H7B	0.1321	0.9550	0.9420	0.096*	
H7C	0.0951	0.8974	1.0219	0.096*	
C8	0.3550 (2)	0.91831 (13)	1.03659 (11)	0.0490 (5)	
H8A	0.4403	0.9306	1.0133	0.059*	
H8B	0.3128	0.9745	1.0497	0.059*	
C9	0.3655 (2)	0.86100 (14)	1.11572 (12)	0.0512 (5)	
H9A	0.4529	0.8626	1.1385	0.061*	
H9B	0.3057	0.8813	1.1594	0.061*	
C10	0.33128 (18)	0.76904 (13)	1.08684 (12)	0.0460 (4)	
Cl1	0.60562 (5)	0.89949 (5)	0.79454 (4)	0.0661 (2)	
Cl2	0.44643 (7)	1.03447 (4)	0.71183 (3)	0.0634 (2)	
N1	0.33425 (15)	0.86043 (9)	0.88631 (9)	0.0376 (3)	
N2	0.28063 (18)	0.77378 (10)	1.00813 (10)	0.0495 (4)	
O1	0.34367 (15)	1.01088 (8)	0.88014 (9)	0.0493 (4)	
O2	0.34379 (18)	0.69963 (12)	1.12676 (10)	0.0677 (5)	

### Atomic displacement parameters (Å^2^)

**Table:**

	*U*^11^	*U*^22^	*U*^33^	*U*^12^	*U*^13^	*U*^23^
C1	0.0509 (10)	0.0420 (9)	0.0347 (8)	−0.0063 (7)	−0.0010 (7)	−0.0023 (7)
C2	0.0419 (8)	0.0325 (8)	0.0325 (8)	−0.0003 (6)	−0.0051 (6)	−0.0013 (6)
C3	0.133 (2)	0.0307 (9)	0.0519 (12)	−0.0056 (11)	0.0152 (13)	−0.0057 (8)
C4	0.177 (3)	0.0556 (14)	0.0523 (14)	−0.0532 (18)	−0.0047 (16)	−0.0051 (10)
C5	0.132 (2)	0.0500 (12)	0.0517 (13)	−0.0423 (14)	−0.0074 (13)	0.0063 (10)
C6	0.0463 (9)	0.0384 (8)	0.0318 (8)	−0.0040 (7)	−0.0051 (6)	0.0021 (6)
C7	0.0486 (11)	0.0909 (17)	0.0534 (12)	0.0083 (11)	0.0025 (9)	0.0066 (11)
C8	0.0672 (12)	0.0430 (9)	0.0367 (9)	−0.0096 (8)	−0.0075 (8)	−0.0038 (7)
C9	0.0591 (11)	0.0615 (12)	0.0330 (9)	−0.0038 (9)	−0.0070 (8)	−0.0004 (8)
C10	0.0472 (9)	0.0549 (11)	0.0359 (9)	0.0012 (8)	0.0030 (7)	0.0098 (8)
Cl1	0.0494 (3)	0.0859 (5)	0.0629 (4)	0.0085 (3)	0.0054 (2)	−0.0065 (3)
Cl2	0.0924 (5)	0.0559 (4)	0.0419 (3)	−0.0146 (3)	0.0005 (2)	0.0120 (2)
N1	0.0490 (8)	0.0311 (7)	0.0328 (7)	−0.0019 (5)	−0.0022 (6)	−0.0014 (5)
N2	0.0691 (10)	0.0393 (8)	0.0400 (8)	−0.0153 (7)	−0.0084 (7)	0.0076 (6)
O1	0.0724 (9)	0.0308 (6)	0.0448 (7)	0.0038 (6)	0.0064 (6)	−0.0010 (5)
O2	0.0858 (11)	0.0644 (10)	0.0528 (9)	0.0071 (8)	0.0007 (8)	0.0255 (7)

### Geometric parameters (Å, º)

**Table:**

C1—C2	1.541 (2)	C6—N2	1.460 (2)
C1—Cl2	1.7647 (19)	C6—N1	1.492 (2)
C1—Cl1	1.780 (2)	C6—C7	1.525 (3)
C1—H1	0.9800	C6—C8	1.546 (2)
C2—O1	1.214 (2)	C7—H7A	0.9600
C2—N1	1.343 (2)	C7—H7B	0.9600
C3—N1	1.464 (2)	C7—H7C	0.9600
C3—C4	1.467 (4)	C8—C9	1.512 (3)
C3—H3A	0.9700	C8—H8A	0.9700
C3—H3B	0.9700	C8—H8B	0.9700
C4—C5	1.511 (4)	C9—C10	1.494 (3)
C4—H4A	0.9700	C9—H9A	0.9700
C4—H4B	0.9700	C9—H9B	0.9700
C5—N2	1.464 (3)	C10—O2	1.221 (2)
C5—H5A	0.9700	C10—N2	1.341 (2)
C5—H5B	0.9700		
			
C2—C1—Cl2	110.76 (12)	N2—C6—C8	102.31 (13)
C2—C1—Cl1	106.84 (12)	N1—C6—C8	111.95 (15)
Cl2—C1—Cl1	110.39 (10)	C7—C6—C8	112.96 (17)
C2—C1—H1	109.6	C6—C7—H7A	109.5
Cl2—C1—H1	109.6	C6—C7—H7B	109.5
Cl1—C1—H1	109.6	H7A—C7—H7B	109.5
O1—C2—N1	124.12 (16)	C6—C7—H7C	109.5
O1—C2—C1	119.90 (15)	H7A—C7—H7C	109.5
N1—C2—C1	115.91 (14)	H7B—C7—H7C	109.5
N1—C3—C4	111.7 (2)	C9—C8—C6	105.61 (15)
N1—C3—H3A	109.3	C9—C8—H8A	110.6
C4—C3—H3A	109.3	C6—C8—H8A	110.6
N1—C3—H3B	109.3	C9—C8—H8B	110.6
C4—C3—H3B	109.3	C6—C8—H8B	110.6
H3A—C3—H3B	107.9	H8A—C8—H8B	108.7
C3—C4—C5	111.1 (2)	C10—C9—C8	105.05 (15)
C3—C4—H4A	109.4	C10—C9—H9A	110.7
C5—C4—H4A	109.4	C8—C9—H9A	110.7
C3—C4—H4B	109.4	C10—C9—H9B	110.7
C5—C4—H4B	109.4	C8—C9—H9B	110.7
H4A—C4—H4B	108.0	H9A—C9—H9B	108.8
N2—C5—C4	109.54 (17)	O2—C10—N2	123.87 (19)
N2—C5—H5A	109.8	O2—C10—C9	127.37 (19)
C4—C5—H5A	109.8	N2—C10—C9	108.75 (15)
N2—C5—H5B	109.8	C2—N1—C3	125.00 (15)
C4—C5—H5B	109.8	C2—N1—C6	117.77 (13)
H5A—C5—H5B	108.2	C3—N1—C6	117.23 (14)
N2—C6—N1	107.07 (14)	C10—N2—C6	114.85 (15)
N2—C6—C7	111.75 (17)	C10—N2—C5	123.23 (16)
N1—C6—C7	110.41 (14)	C6—N2—C5	121.90 (16)
			
Cl2—C1—C2—O1	−17.6 (2)	N2—C6—N1—C2	−164.27 (15)
Cl1—C1—C2—O1	102.68 (17)	C7—C6—N1—C2	73.9 (2)
Cl2—C1—C2—N1	165.39 (13)	C8—C6—N1—C2	−52.9 (2)
Cl1—C1—C2—N1	−74.34 (16)	N2—C6—N1—C3	15.0 (2)
N1—C3—C4—C5	−62.4 (4)	C7—C6—N1—C3	−106.9 (2)
C3—C4—C5—N2	28.4 (4)	C8—C6—N1—C3	126.3 (2)
N2—C6—C8—C9	−17.3 (2)	O2—C10—N2—C6	179.24 (19)
N1—C6—C8—C9	−131.65 (17)	C9—C10—N2—C6	0.3 (2)
C7—C6—C8—C9	103.0 (2)	O2—C10—N2—C5	0.7 (3)
C6—C8—C9—C10	17.9 (2)	C9—C10—N2—C5	−178.2 (2)
C8—C9—C10—O2	169.3 (2)	N1—C6—N2—C10	128.79 (17)
C8—C9—C10—N2	−11.8 (2)	C7—C6—N2—C10	−110.2 (2)
O1—C2—N1—C3	176.6 (2)	C8—C6—N2—C10	10.9 (2)
C1—C2—N1—C3	−6.5 (3)	N1—C6—N2—C5	−52.6 (3)
O1—C2—N1—C6	−4.3 (3)	C7—C6—N2—C5	68.4 (3)
C1—C2—N1—C6	172.63 (14)	C8—C6—N2—C5	−170.5 (2)
C4—C3—N1—C2	−142.1 (2)	C4—C5—N2—C10	−151.3 (3)
C4—C3—N1—C6	38.8 (3)	C4—C5—N2—C6	30.2 (4)

### Hydrogen-bond geometry (Å, º)

**Table:**

*D*—H···*A*	*D*—H	H···*A*	*D*···*A*	*D*—H···*A*
C1—H1···O2^i^	0.98	2.15	3.115 (2)	168
C3—H3*B*···O2^i^	0.97	2.55	3.502 (3)	169

Symmetry code: (i) *x*, −*y*+3/2, *z*−1/2.
